# Evaluating diurnal rhythms of host responses to enteric norovirus infection in mouse models

**DOI:** 10.1097/IN9.0000000000000052

**Published:** 2024-12-03

**Authors:** Jianglin Zhang, Robert C. Orchard, Zheng Kuang

**Affiliations:** 1 Department of Biological Sciences, Carnegie Mellon University, Pittsburgh, PA, USA; 2 Department of Immunology, University of Texas Southwestern Medical Center, Dallas, TX, USA

**Keywords:** circadian rhythms, norovirus, infection, mucosa, immune

## Abstract

Norovirus is a leading cause of gastroenteritis worldwide. The factors required for the life cycle and pathogenesis of norovirus in humans remain unclear. Mouse models of norovirus infection have been widely used to explore the crosstalk between norovirus and the host. The circadian clock entrains biological processes and behaviors including eating and sleeping in response to day–night cycles. How the mucosal immunity is diurnally programmed in response to norovirus infection remains largely unknown. Here, we provide procedures for preparing a murine norovirus strain CR6 and for infection in mouse models under normal day/night light cycles or jet-lag conditions. We also present procedures to quantify viral copies and antiviral response transcripts in host tissues. These protocols will help facilitate studies of norovirus infection and immunometabolic responses from the circadian perspective.

## 1. Introduction

Norovirus is the most common cause of non-bacterial acute gastroenteritis worldwide. About 200,000 deaths are estimated to be caused by norovirus infection ^[1]^ and a tremendous financial burden of up to $60 million is contributed by the norovirus outbreak annually ^[2]^. Human norovirus, which comprises around 30 strains, outbreaks among people of different ages, especially in children and elderly people. However, it is still challenging to study human norovirus in the absence of effective animal models ^[3]^. So far, the biology of human norovirus is not well understood. Murine norovirus (MNoV) is a genogroup V enteric norovirus, including strains CR6 and CW3. Mouse models of MNoV infection are well-established to understand the replication and pathogenesis of norovirus in a natural host.

The mammalian circadian system consists of the core clock transcription factors including BAML1, CLOCK, PER, CRY, and REV-ERB ^[4]^. A variety of intestinal processes such as nutrient absorption and mucosal immunity are synchronized by the circadian clock and display 24-hour diurnal rhythms. Interruption of these rhythms disrupts the immunometabolic homeostasis in the gut and leads to various diseases ^[5–7]^. For example, the ablation of BAML1 in group 3 innate lymphoid cells (ILC3) impairs the gut ILC3 homeostasis and intestinal epithelial reactivity, which disrupts lipid metabolism and increases susceptibility to intestinal infection ^[8]^. The circadian clock also regulates the toll-like receptors (TLRs) signaling in macrophages and programs the polarization state and metabolic conditions to affect immune function regulation ^[9]^. Intriguingly, microbial burden and foodborne pathogens show diurnal rhythms coupled with the feeding schedule ^[10,11]^. Accordingly, the rhythmic expression of TLR4 and major histocompatibility complex class II in intestinal epithelium is regulated by the gut microbiota to maintain the gut homeostasis ^[12,13]^. Intestinal epithelial cells also rhythmically express antimicrobial proteins, which contributes to the diurnal rhythms of antibacterial immunity in the intestine ^[11]^. It remains unknown how mucosal immunometabolism is diurnally programmed in response to fluctuating risks of norovirus infection in the intestine. Therefore, developing a diurnal norovirus infection model in mice is critical for understanding circadian rhythms in norovirus infection.

In this article, we first describe a method on how to produce murine norovirus CR6 (MNoV^CR6^) strain (basic protocol 1 and support protocols 1–3). Basic protocol 2 describes diurnal infection and quantification of MNoV in mice using the MNoV^CR6^ strain. The diurnal rhythm of immune responses to the MNoV^CR6^ infection is determined by analyzing the expression of interferon pathway genes (basic protocol 2).

## 2. Equipment

CO_2_ Incubator (Thermo Fisher Scientific, HERAcell vios 160i, Waltham, MA, USA); Centrifuge (Beckman Coulter, Allegra X-30R, Brea, CA, USA); Centrifuge 5425R (Eppendorf, Enfield, CT, USA); refrigerated (Eppendorf, Enfield, CT, USA); Pipette (Gilson Pipetman, Madison, WI, USA); Circadian box (Actimetrics, Wilmette, IL, USA); Excella E24 Incubator Shaker (Eppendorf, model: New Brunswick, Enfield, CT, USA); NanoDrop One C (Thermo Fisher Scientific, Waltham, MA, USA); Biosafety cabinet (Thermo Fisher Scientific, model 1375, Waltham, MA, USA); Thermomixer F1.5 (Eppendorf, Enfield, CT, USA); Magnetic stir bar (Fisher Scientific, 1451352, Waltham, MA, USA); −86 °C Ultra-Low Temperature Freezer (Thermo Fisher Scientific, Waltham, MA, USA); FastPrep-24™ 5G bead beating grinder and lysis system (MP Biomedicals, SKU 116005500, Santa Ana, CA, USA).

## 3. Ethical statement

All experiments were performed using protocols approved by the Institutional Biosafety Committee Recombinant or Synthetic Nucleic Acid Molecules Research Application (rDNA080223, August 2, 2023) and the Institutional Animal Care and Use Committee of Carnegie Mellon University (PROTO202000017, June 20, 2024). This study adhered to the Helsinki Declaration and was in compliance with the Animal Research: Reporting of In Vivo Experiments (ARRIVE) guidelines.

## 4. Basic protocol 1: preparation of MNoV^CR6^

### 4.1 Materials and reagents

HEK293T cell line (Lora V. Hooper Laboratory, Dallas, TX, USA; received on July 1, 2021); BV2 cell line (Robert C. Orchard Laboratory, Dallas, TX, USA; received on July 1, 2021); DMEM-GlutaMax (Gibco, Catalog Number: 10569044, Miami, FL, USA); minimum essential medium (MEM) (Corning, Catalog Number: 15-010-CM, Corning, NY, USA); Penicillin-Streptomycin (100×) (Gibco, Catalog Number: 15140122, Miami, FL, USA); pMNoV^CR6^ (see Support protocol 2); Lipofectamine 3000 (Invitrogen, Catalog Number: L3000015, Waltham, MA, USA); Dulbecco’s Phosphate-Buffered Saline (1× DPBS, Gibco, Catalog Number: 14190250, Miami, FL, USA); 6-well tissue culture plate (Celltreat, Catalog Number: 229105, Pepperell, MA, USA); 10-cm cell culture dish (Greiner Bio-One, Catalog Number: 07000386, Frickenhausen, Germany); Syringe Filters (0.22 μM polyvinylidene fluoride [PVDF], Celltreat, Catalog Number: 229751, Pepperell, MA, USA); Protein concentrator (100 Kda PES, Thermo Fisher Scientific, Catalog Number: PI88533, Waltham, MA, USA).

### 4.2 Procedure

(1) On day 1, seed HEK293T cells in a 6-well plate (5 × 10^5^ cells/well) and incubate cells in a CO_2_ incubator at 37 °C and 5% CO_2_ for 24 hours.
(2) On day 2, when HEK293T cells reach 80%~90% confluency, transfect cells with a plasmid (2 μg) containing MNoV^CR6^ viral genomes (pMNoV^CR6^) using Lipofectamine 3000 following the manufacturer’s instruction. One well can be transfected with a green fluorescent protein (GFP) plasmid as the negative control.

Note: Optimization may be performed according to the manufacturer’s instruction. Alternative transfection kits and protocols that work for HEK293T cells such as calcium phosphate transfection ^[14]^ can also be used for pMNoV^CR6^ transfection.

(3) After 48 hours of transfection, check the health of cells and GFP expression of the control well. Then collect cells and medium and freeze at −80 °C and thaw for 3 times to lyse cells and release the virus.
(4) Spin the cell lysate medium at 1000 × *g* for 10 minutes at 4 °C.
(5) Collect the supernatants and aliquot them. This is now the p0 (passage 0) of the virus. Store the virus at −80 °C, or proceed to the next step concurrently with this one.
(6) To generate p1 stocks, seed BV2 cells in a 10-cm cell culture dish at a 1:3 split ratio, and culture with 10 mL DMEM-GlutaMax supplemented with 10% fetal bovine serum (FBS) and 1% penicillin and streptomycin overnight. When BV2 cells reach approximately 90% confluency, infect cells with p0 virus (0.05 MOI) and culture cells in the incubator. This is the day 1 for BV2 cell infection. For the control group, add the medium from the control transfection group (GFP plasmid). Cells will remain healthy.
(7) From day 2 to day 5, check cells until you see about 50% cytopathic effect (CPE). This sign suggests that the virus is actively replicating and causes cell death.

Note: If cells are not dying after 5 days, virus production in BV2 cells is not successful and the cells should be abandoned.

(8) Collect all the cells and media when the culture reaches to 80%–90% CPE. Freeze at −80 °C and thaw for 3 times to break the cells and release the virus.
(9) Spin the cell lysate medium at 1000 × *g* for 10 minutes at 4 °C. Filter the supernatants through a 0.22 μM PVDF membrane. Aliquot the virus (500 μL/tube) in 1.5 mL Eppendorf tubes and store them at −80 °C for future use (the virus titer is about 10^7^ plaque-forming units [PFU]/mL). To obtain a higher virus titer, the filtered supernatants can be concentrated with a protein concentrator at 4000 rpm following the manufacturer’s instruction (the virus titer should be approximately 1–5 × 10^8^ PFU/mL after concentrating). The concentrated virus was also aliquoted in 1.5 mL Eppendorf tubes (30 μL/tube) and store them at −80 °C for future use.
(10) Optional, to avoid potential side effects from the cell culture medium such as antibiotics and FBS, the cell culture medium could be switched to 1× phosphate buffered saline (PBS) during the concentration process.
(11) The virus titer in the frozen stock can be determined by the plaque assay (see Support protocol 1).

## 5. Basic protocol 2: the diurnal rhythm of MNoV infection in mice

### 5.1 Materials and reagents

MNoV^CR6^ virus (see Basic protocol 1); DMEM-GlutaMax (Gibco, Catalog Number: 10569044, Miami, FL, USA); fetal bovine serum (GeminiBio, Catalog Number: 100-106, Sacramento, CA, USA); C57BL/6 wild-type mice (The Jackson laboratory, Bar Harbor, ME, USA); 1.5 mL Eppendorf tube (Bio Plas, 4030, San Rafael, CA, USA); 15 mL polypropylene centrifuge tube (Celltreat, 667015B, Pepperell, MA, USA); 2 mL capped screw tube (Bio Plas, 4203, San Rafael, CA, USA); scissors (Midland Scientific, WSI 310-045, La Vista, NE, USA); Graefe forceps (Fine Science Tools, 1105110, Foster, CA, USA); disruption beads for tissue (RPI, SKU 9832, Mount Prospect, IL, USA); isopropanol (Thermo Fisher Scientific, AC149320025, Waltham, MA, USA); chloroform (Thermo Fisher Scientific, AC423550040, Waltham, MA, USA); ethanol (Thermo Fisher Scientific, AC615090040, Waltham, MA, USA); reverse transcription kit (Thermo Fisher Scientific, FERK1691, Waltham, MA, USA); EvaGreen qPCR Master Mix (Biotium, 31045, Fremont, CA, USA); pMNoV^CR6^ (see support protocol 2); Tri reagent (Sigma-Aldrich, T9242, St. Louis, MO, USA).

### 5.2 Procedure

**Figure 1.**
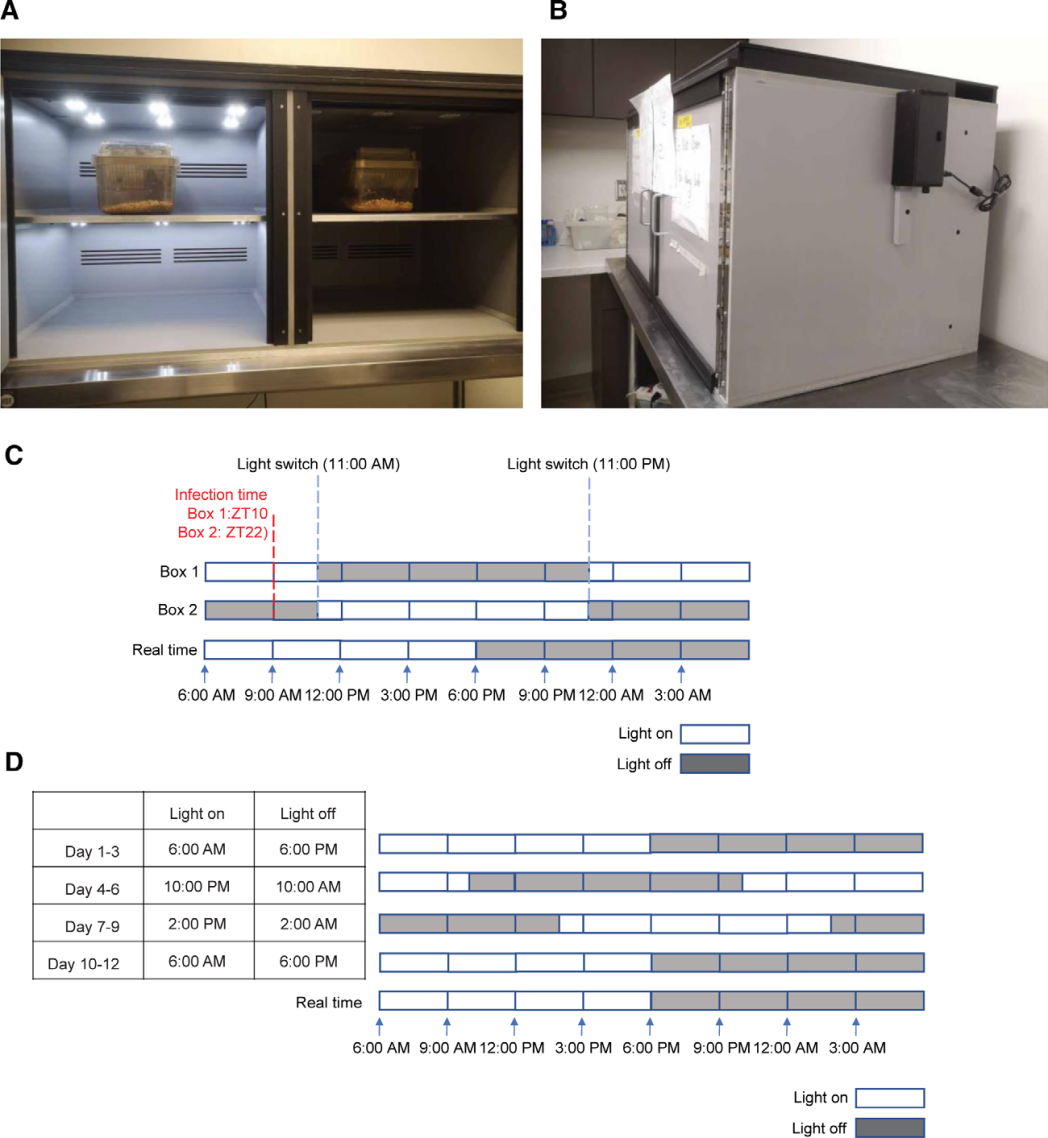
**Examples of the circadian boxes and light schedule setup.** The face view (A) and the side view (B) of the circadian boxes. As shown in (A), the left side is light on, while the right side is light off. This indicates that mice in the left box are under daytime, whereas mice in the right box are under nighttime. (B) The side outline view of the circadian box. (C) An example of the light schedule setup in the circadian boxes. 9 am represents ZT10 in box 1 and ZT22 in box 2. (D) An example of the jet-lag setup using a circadian box. The light schedule is maintained for 3 days and then the light is turned on 8 hours earlier. The new light schedule is maintained for another 3 days and shifted by 8 hours again.

Note: Circadian boxes are ventilated, light-tight boxes where internal lights can be controlled to be on and off at specific times. In a typical circadian experiment, the light/dark cycle is set to be 12 hours of light and 12 hours of darkness. We define “zeitgeber time” (ZT) as the time relative to this experimental light/dark cycle. The light-on time is equal to the sunrise time, which is ZT0, and the light-off time is equal to the sunset time, which is ZT12. Therefore, if the plan is to infect mice at ZT10 and ZT22 at 9:00 am in real time, light can be programmed to be on at nighttime (ie, 11:00 pm) and turned off at daytime (ie, 11:00 am) in the box 1. In the box 2, the light can be programmed to be on at daytime (ie, 11:00 am) and off at nighttime (ie, 11:00 pm) (Figure 1C). Therefore, the time in the box 1 is ZT10 and the time in the box 2 is ZT22 at 9:00 am. Mice can be sacrificed at 9 am the next day to collect samples for a 24-hour infection experiment. The light schedules, infection, and sample collection times can be adjusted for different experimental designs.

(1) The light/dark control in the circadian box (Figure 1A, B) is set up with two opposite time schedules so that samples with a 12-hour interval can be collected at the same time.

Optional: to study whether diurnal infection of norovirus in mice can be interrupted under jet-lag conditions, an experimental jet lag can be introduced using circadian boxes (Figure 1D). Specifically, a light schedule of 12-hour light on and 12-hour light off is initiated in the box. For every 3 days, the light will be turned on 8 hours earlier than the previous setting. Accordingly, the light will be turned off also 8 hours earlier. Therefore, the schedule of 12-hour light on and 12-hour light off is maintained the same. For the control group, the light/dark schedule is left unchanged. After four rounds of jet-lag interruption, mice will be infected with a virus as described later and the light schedule will continue to be shifted every 3 days for the duration of the experiment.

If light boxes are not available, mice can be housed in a regular mouse holding room with a light schedule of 12 hours of light and 12 hours of darkness. Infections can be done at different time points according to the experimental design. For example, if the light is turned on at 6:00 am and infection is designed to occur at ZT2, then infection should be performed at 8:00 am.

(2) Cohouse 10 to 20 gender-matched C57BL/6 mice at 4 to 6 weeks old (five mice/cage) and move the mice to the circadian boxes for at least 2 weeks. Mice should be randomly assigned for the experiment without investigator blinding. This will allow the mice to adapt to the light/dark schedules.

Note: As the difference of gut microbiotas may affect virus infection, it is good to cohouse mice before infection. According to our experience, to achieve sufficient statistical power, at least five mice per group are recommended to start an experiment. Therefore, 10 to 20 mice are required for a two-time-point infection experiment. The number of mice needs to be optimized for each specific experiment. It is recommended to follow the Animal Research: Reporting of In Vivo Experiments guidelines (https://arriveguidelines.org/sites/arrive/files/documents/ARRIVE%20guidelines%202.0%20-%20English.pdf) to design mouse experiments, for example, sample size, inclusion, and exclusion criteria.

(3) Mice under normal light cycle or under jet lag are orally infected with MNoV^CR6^ diluted in DMEM-GlutaMax using a 200 μL pipette at daytime (eg, ZT4) and nighttime (eg, ZT16).

Note: Dose and infection time can be optimized based on experimental design. 10^6^ PFU/mouse in 20 μL DMEM-GlutaMax is commonly used and is recommended as the starting point. Acute infection can be monitored (1–7 days) or chronic infection (21 days or longer) depending upon the MNoV strain ^[15]^ by quantitative reverse transcription polymerase chain reaction (qRT-PCR) with the tissue samples.

(4) Mice are euthanized with CO_2_ and tissues including small intestine, large intestine, mesenteric lymph nodes and spleen are flash frozen in liquid nitrogen and stored in a −80 °C freezer.

Note: Small intestine and large intestine are flushed with cold 1× PBS to remove luminal contents. About 0.5 to 1 cm of the intestinal tissue is sufficient for downstream viral copy quantification.

(5) Move tissues into 2 mL autoclaved tubes with the disruption beads. Add 500 μL TRI reagent and homogenize tissues using the FastPrep-24™ 5G. Extract total RNA from the homogenized tissue using the Direct-zol™ RNA Purification Kit according to the manufacture’s instruction.

Note: Equivalent bead beater or tissue homogenizer can be used to homogenize tissues. Equivalent RNA extraction kits or protocols can be used to purify RNA.

(6) The concentration of total RNA is determined by NanoDrop. About 0.5 to 1 μg total RNA is used for reverse transcription with the reverse transcription kit to generate cDNA.

Note: Equivalent reverse transcription reagent can be used to generate cDNA. It is recommended to use the same amount of total RNA as input.

**Figure 2.**
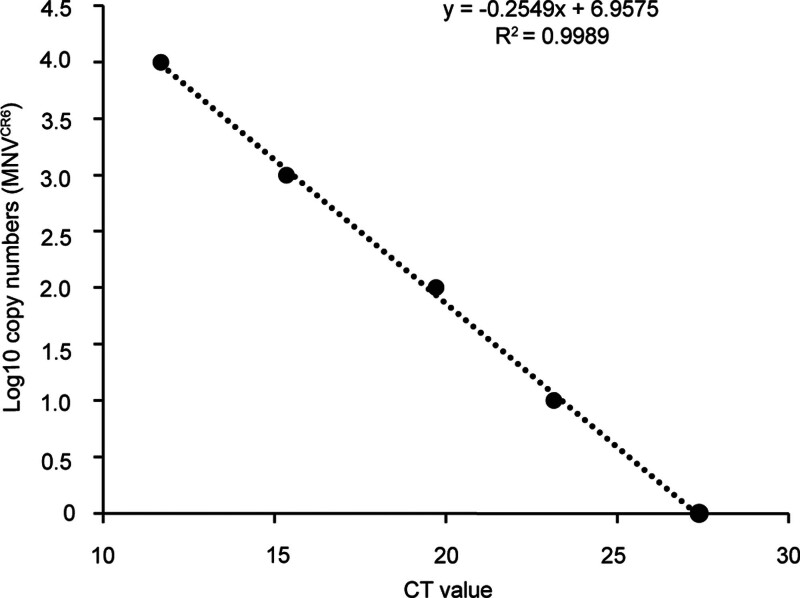
**An example of the standard curve for estimating MNoV**^CR6^ **genomic copy numbers by qRT-PCR using pMNoV**^CR6^. MNoVCR6, murine norovirus CR6; pMNoV^CR6^, plasmid (2 μg) containing MNoV^CR6^ viral genomes. qRT-PCR, quantitative reverse transcription polymerase chain reaction.

Note: We recently showed that when mice were infected with MNoV^CR6^ at either daytime or nighttime, the genome copy number was significantly higher at ZT10 than ZT22 in wild-type mice ^[16]^. This finding indicates the presence of diurnal rhythms in norovirus infection.

(7) To quantify viral genomic copy numbers in tissues, qRT-PCR is performed with the cDNA generated from step 6 using the primers, MNoV_quantF and MNov_quantR (Table 1). The pMNoV^CR6^ (see Support protocol 2) is used as the template for generating the standard curve (Figure 2). Later we provide an example to illustrate how to generate a standard. 10^7^ copies/mL of pMNoV^CR6^ is diluted by 10 folds with ddH_2_O to generate a series of concentrations as follows: 10^7^, 10^6^, 10^5^, 10^4^, 10^3^. 3.5 μL of each standard is added into qPCR reactions. To calculate the viral copy numbers, use the formula: log10 (3.5 × 10^7^/1000). A series of log10 copy numbers are 4.544, 3.544, 2.544, 1.544, and 0.544, which are set as *y* axis, *x* axis is the qPCR-determined CT values, as shown in Figure 2. 3.5 can be replaced with other values if a different volume of cDNA standards is added to the qPCR reactions. To determine the expression level of interferon genes in these tissues, qRT-PCR is performed with cDNA from step 6 using primers listed in Table 1. *Gapdh* can be used as a reference.

**Table 1:** qRT-PCR primers.

Gene name	3’-5’ sequence
MNoV_quantF	CACGCCACCGATCTGTTCTG
MNoV_quantR	GCGCTGCGCCATCACTC
*mIfna_*F	GGACTTTGGATTCCCGCAGGAGAAG
*mIfna_*R	GCTGCATCAGACAGCCTTGCAGGTC
*mIfnb_*F	AACCTCACCTACAGGGCGGACTTCA
*mIfnb_*R	TCCCACGTCAATCTTTCCTCTTGCTTT
*mIfnl2/3_*F	AGTGGAAGCAAAGGATTG
*mIfnl2/3_*R	GAGATGAGGTGGGAACTG
*mIfnlr1_*F	GACGAGTACAGGCAGCTTCC
*mIfnlr1_*R	AGCATTGACCCTTAGGATCTTCTC
*mIfnar1_*F	CATGTGTGCTTCCCACCACT
*mIfnar1_*R	TGGAATAGTTGCCCGAGTCC
*mIfnar2_*F	CTATCGTAATGCTGAAACGG
*mIfnar2_*R	CGTAATTCCACAGTCTCTTCT
*mMx1_*F	TCTGAGGAGAGCCAGACGAT
*mMx1_*R	ACTCTGGTCCCCAATGACAG
*mOasi2_*F	GGATGCTGGGAGAGAATCG
*mOasi2_*R	TCGCCTGCTCTTCGAAACTG
*mIsg20_*F	CCATGGACTGTGAGATGGTG
*mIsg20_*R	CTCGGGTCGGATGTACTTGT
*mIsg15_*F	CAGGACGGTCTTACCCTTTCC
*mIsg15_*R	AGGCTCGCTGCAGTTCTGTAC
*Gapdh_*F	TGGCAAAGTGGAGATTGTTGCC
*Gapdh_*R	AAGATGGTGATGGGCTTCCCG

qRT-PCR, quantitative reverse transcription polymerase chain reaction.

## 6. Support protocol 1: preparation of the plasmid containing the MNoV^CR6^ genome for the standard

### 6.1 Materials and reagents

The *E. coli* strain containing the pMNoV^CR6^ (10,894 bp) can be obtained from the Robert C. Orchard Laboratory. The plasmid is under a material transfer agreement (MTA) with Washington University in St. Louis and the MTA can be obtained via emailing to mta@wustl.edu. Tryptone (Fisher Scientific, OXLP0042B, Waltham, MA, USA); sodium chloride (Fisher Scientific, BP358-1, Waltham, MA, USA); yeast extract (Thermo Fisher Scientific, LP0021B, Waltham, MA, USA); agar (Sigma-Aldrich, A1296, St. Louis, MO, USA); 1.5 mL microcentrifuge tube (Bio Plas, 4030, San Rafael, CA, USA); petri dish (Fisher Scientific, FB0875712, Waltham, MA, USA); penicillin (Thermo Fisher Scientific, 15140122, Waltham, MA, USA); inoculation loop (VWR, 12000806, Radnor, PA, USA); plasmid extraction kit (Zymo, D4020, Irvine, CA, USA).

### 6.2 Procedure

(1) Recover the *E. coli* strain containing the pMNoV^CR6^ plasmid on an Luria Broth (LB) agar plate supplemented with ampicillin, and culture the plate in a microbiological incubator at 37 °C overnight.
(2) Pick up a single colony and inoculate it in 2 mL LB medium supplemented with 50 μg/mL ampicillin, which will be cultured at 37 °C with shaking at 200 rpm overnight.
(3) Extract the plasmid by using the Zymo plasmid extraction kit.

Note: Alternative plasmid extraction kits or protocols can be used.

(4) Measure the plasmid concentration using NanoDrop or a similar instrument and estimate the copy number in the plasmid stock. The following website is one tool that can be used to estimate viral copy (https://www.technologynetworks.com/tn/tools/copynumbercalculator). A stock of 117.59 ng/μL is 1 × 10^10^ copies/μL.

Note: Number of copies per μL = (DNA concentration [ng/μL] × [6.022 × 10^23^])/(length of template (bp) × [1 × 10^9^] × 650).

(5) Store the plasmid at a −20 °C freezer for generating the standard to quantify the virus copy number in qRT-PCR analysis.

## 7. Support protocol 2: determine virus titer with the plaque assay

### 7.1 Materials and reagents

MNoV^CR6^ virus (see Basic protocol 1); BV2 cell line (Robert C. Orchard Laboratory; July 1, 2021); fetal bovine serum (GeminiBio, 100-106, Sacramento, CA, USA); DMEM-GlutaMax (Gibco, 10569044, Miami, FL, USA); Dulbecco’s phosphate-buffered saline (1× DPBS, Gibco, 14190250, Miami, FL, USA); 1% methylcellulose MEM (see Support protocol 3); 4% formaldehyde in 1× PBS: add 5.4 mL of 37% formaldehyde solution (Sigma-Aldrich, 252549, St. Louis, MO, USA) in 44.6 mL of 1× PBS; 0.1% crystal violet staining buffer: mix 280 mL of water, 40 mL of 1% crystal violet (Sigma-Aldrich, V5265, St. Louis, MO, USA), and 80 mL of 95% ethanol together; 6-well tissue culture plate (Celltreat, 229105, Pepperell, MA, USA); 10-cm cell culture dish (Greiner Bio-One, 07000386, Frickenhausen, Germany); beaker (Fisher Scientific, 10-210-685, Waltham, MA, USA).

### 7.2 Procedure

(1) Seed BV2 cells (~5 × 10^5^/well) in a 6-well plate and incubate cells in the incubator at 37 °C and 5% CO_2_.
(2) When cells reach ~80% confluency after approximately 24 hours, remove the medium from the 6-well plate. Add 200 μL MEM to each well.
(3) Prepare a series dilution of MNoV^CR6^ standard from the stock in a 10-fold manner with MEM. For example, add 25 μL from the previous higher-concentration stock into 225 μL of MEM for five consecutive times. This will generate 225 μL each of the following standards, 10^0^, 10^−1,^ 10^−2^, 10^−3^, 10^−4^, 10^−5^.

Note: Tips should be changed in between each dilution to eliminate carryover. Dilution schemes may vary depending on the desired limit of detection.

(4) Starting from the lowest concentration, add 200 μL of diluted virus to each well, and mix it by gently rocking the plate.
(5) Incubate the plate at room temperature and gently rock it to mix medium every 20 minutes for up to 1 hour.

Note: Gently rocking during incubation could increase the infection efficiency.

(6) After 1 hour, remove the medium from the plate. Add 2 mL of 1% methylcellulose MEM in each well and mix the medium by gentle rocking.
(7) Incubate the plate in the 37 °C CO_2_ incubator for 48 to 72 hours.

Note: 3 days for larger and easily distinguished CR6 plaque.

(8) Remove the media and then add 2 mL of fixation buffer (4% formaldehyde in 1× PBS) and incubate for 5 to 10 minutes at room temperature to fix the cells.
(9) Dump the liquid in a beaker. Add 2 mL of staining buffer in each well and incubate for at least 1 hour or overnight/over the weekend.
(10) Dump the liquid from the wells and wash the cells with water. Dry the plate at room temperature and count the plaques.

Note: Choosing the suitable dilution to count the plaque number is important to get an accurate estimation of virus concentration. Plaques should be visible and easily distinguished from each other. Viral stock concentration can be calculated by simple mathematical methods. For example, if 30 plaques are detected at the 10^−3^ dilution when 200 μL viral standard is added, the stock concentration is 30 × 10^3^/0.2 = 1.5 × 10^5^ PFU/mL.

## 8. Support protocol 3: preparation of 1% methylcellulose

### 8.1 Materials and reagents

Methylcellulose (Sigma-Aldrich, Catalog Number: M0387, St. Louis, MO, USA); 1 L reusable media storage bottle (Fisher Scientific, FB8001000); Minimum Essential Medium (Corning, Catalog Number: MT15010CM, Corning, NY, USA); fetal bovine serum (GeminiBio, Catalog Number: 100-106, Sacramento, CA, USA); magnetic stir bar (Fisher scientific, 1451352, Waltham, MA, USA).

### 8.2 Procedure

(1) Autoclave 10 g of methyl cellulose dry powder in a 1 L bottle with a magnetic stir bar.

Note: The stir bar and bottle should never see detergent.

(2) Keep the bottle hot or warm in a 55 °C water bath. Slowly add 800 mL 37 °C prewarmed MEM media in the bottle in a biosafety cabinet. Shake the bottle hard by hand.
(3) Put the bottle on a heater at 37 °C and stir the media for 3 hours to let methyl cellulose powder suspended.
(4) Move the bottle to 4 °C and stir overnight until the solution becomes transparent. Otherwise, the medium should be discarded if it does not become transparent.

Note: The solubility of methyl cellulose is very temperature sensitive. Please make sure the temperature for the overnight stir process is approximately 4 °C, especially if the solution does not become transparent.

(5) Add 100 mL FBS, 10 mL l-glutamine, and 10 mL HEPES to the 800 mL MEM methylcellulose medium and mix it well by shaking. Fill to ~ 1 L with MEM and make sure the methylcellulose is well dispersed. Store it at 4 °C for future use.

Note: Make sure MEM has glutamine to support the growth of BV2 cells.

## 9. Critical parameters and troubleshooting

### 9.1 Produce MNoV^CR6^

(1) Make sure cells are in good condition and the cell density is measured accurately. These can affect the virus production titer.
(2) The transfection efficiency affects virus production titer. A GFP-positive empty plasmid can be cotransfected to measure the transfection efficiency.

### 9.2 The diurnal rhythm of MNoV infection in mice

(1) When comparing different genetic mouse groups, mice should be cohoused 2 to 4 weeks before infection.
(2) In the protocol, we use C57BL/6 male mice to examine the diurnal rhythms in MNoV^CR6^ infection and host response. It would be interesting to examine other mouse models such as female mice and genetic mutant mice and other norovirus strains such as MNoV^CW3^. The infection time points in a day/night cycle and infection time length could be optimized. For example, to determine the replication of MNoV^CW3^ in the host, the infection time length could be optimized to 3 days, as MNoV^CW3^ is an acute infection virus ^[17]^.
(3) Make sure the luminal contents in the intestine are cleaned clearly, as residual norovirus in the luminal contents may interfere with the quantification of norovirus copy numbers in the intestinal tissues.

## 10. Discussion and conclusions

Irregular working schedules and travel across time zones rapidly have become prevalent nowadays, which disrupt circadian rhythms. Misalignment of circadian rhythms affects the immune system and metabolism ^[11,18]^. For example, the rhythmicity of leukocytes and immature hematopoietic cell numbers are lost in the *Bmal1*^−/−^ mice ^[19,20]^. Intriguingly, circadian rhythms in the immune system are critical for the host to fight against diurnal challenges of foodborne viruses ^[21]^. We describe a diurnal infection of MNoV in mice to study the role of circadian rhythms in host immune responses against norovirus infection and a jet-lag protocol to determine whether circadian interruption affects the immune response to norovirus infection.

Interferon-stimulated genes (ISGs) exert key functions in antiviral responses. Hundreds of ISGs are induced by interferons (IFNs), a family of cytokines produced in response to infection. There are three types of IFNs: type I IFNs (IFN-αs, IFN-β, others), type II IFN (IFN-γ), and type III IFNs (IFN-λs), which are differentiated by receptor usage. Interferons have been reported to prevent systemic spread of MNoV and control persistent enteric infection ^[22]^. How the expression of interferons is diurnally regulated in response to norovirus infection remains less understood. Furthermore, different MNoV strains such as MNoV^CR6^ and MNoV^CW3^ displayed distinct pathogenic properties ^[17]^. MNoV^CR6^ induces persistent infection in the intestine and can be detected in MLNs, while MNoV^CW3^ causes systemically acute infection by initiating infection in the small intestine and spreads through the large intestine, spleen, and MLNs. Therefore, the diurnal rhythm of immunity in mice in response to MNoV^CR6^ and MNoV^CW3^ may exhibit different characteristics.

The production of interferon is strain background dependent during cytomegalovirus infection ^[23]^. Geist and Hinde also showed a higher total bronchoalveolar lavage (BAL) and BAL lymphocyte count in C57BL/6 mice after cytomegalovirus infection compared with BALB/cJ mice ^[24]^. The burden of MNoV^CR6^ is reduced in BALB/cJ mice compared with C57BL/6 mice, due to the genetic background difference between BALB/cJ and C57BL/6 mice ^[25]^. The genetic backgrounds between different mouse strains should also be considered when studying the rhythmicity of norovirus infection. Together, we describe a series of protocols to study the diurnal rhythms of immune responses against norovirus infection and discuss potential factors that contribute to the diurnal rhythms of norovirus infection in the host.

## Author contributions

Conceptualization, methodology, investigation, visualization, project administration, supervision, and writing-original draft: J.Z., Z.K.; Funding acquisition and resources: Z.K.; Writing-review & editing: J.Z., R.C.O., Z.K.

## Conflicts of interest

The authors declare that they have no conflict of interest.

## Funding

This work was supported by National Institutes of Health grant R00DK120897 (Z.K.), National Institutes of Health grant DP2DK136278 (Z.K.), and the Shurl and Kay Curci Foundation (Z.K.).

## References

[1] Lopman BA Steele D Kirkwood CD. The vast and varied global burden of norovirus: prospects for prevention and control. PLoS Med. 2016;13(4):e1001999. 27115709 10.1371/journal.pmed.1001999 PMC4846155

[2] Cates JE Vinjé J Parashar U. Recent advances in human norovirus research and implications for candidate vaccines. Expert Rev Vaccines. 2020;19(6):539-48. 32500763 10.1080/14760584.2020.1777860 PMC10760411

[3] Strong DW Thackray LB Smith TJ. Protruding domain of capsid protein is necessary and sufficient to determine murine norovirus replication and pathogenesis in vivo. J Virol. 2012;86(6):2950-8. 22258242 10.1128/JVI.07038-11 PMC3302348

[4] Scheiermann C Gibbs J Ince L. Clocking in to immunity. Nat Rev Immunol. 2018;18(7):423-37. 29662121 10.1038/s41577-018-0008-4

[5] Brooks JF Hooper LV. Interactions among microbes, the immune system, and the circadian clock. Semin Immunopathol. 2020;42(6):697-708. 33033938 10.1007/s00281-020-00820-1

[6] Gargari BP Namazi N Khalili M. Is there any place for resistant starch, as alimentary prebiotic, for patients with type 2 diabetes? Complement Ther Med. 2015;23(6):810-5. 26645521 10.1016/j.ctim.2015.09.005

[7] Sharma SA Oladejo SO Kuang Z. Chemical interplay between gut microbiota and epigenetics: implications in circadian biology. Cell Chem Biol. 2024; S2451-9456(24):00178-8. 10.1016/j.chembiol.2024.04.016 PMC11569273 38776923

[8] Godinho-Silva C Domingues RG Rendas M. Light-entrained and brain-tuned circadian circuits regulate ILC3s and gut homeostasis. Nature. 2019;574(7777):254-8. 31534216 10.1038/s41586-019-1579-3 PMC6788927

[9] Zeng Y Guo Z Wu M. Circadian rhythm regulates the function of immune cells and participates in the development of tumors. Cell Death Discov. 2024;10(1):199. 38678017 10.1038/s41420-024-01960-1 PMC11055927

[10] Thaiss CA Levy M Korem T. Microbiota diurnal rhythmicity programs host transcriptome oscillations. Cell. 2016;167(6):1495-510.e12. 27912059 10.1016/j.cell.2016.11.003

[11] Brooks JF II Behrendt CL Ruhn KA. The microbiota coordinates diurnal rhythms in innate immunity with the circadian clock. Cell. 2021;184(16):4154-67.e12. 34324837 10.1016/j.cell.2021.07.001 PMC8967342

[12] Mukherji A Kobiita A Ye T. Homeostasis in intestinal epithelium is orchestrated by the circadian clock and microbiota cues transduced by TLRs. Cell. 2013;153(4):812-27. 23663780 10.1016/j.cell.2013.04.020

[13] Tuganbaev T Mor U Bashiardes S. Diet diurnally regulates small intestinal microbiome-epithelial-immune homeostasis and enteritis. Cell. 2020;182(6):1441-59.e21. 32888430 10.1016/j.cell.2020.08.027

[14] Kingston RE Chen CA Rose JK. Calcium phosphate transfection. Curr Protoc Mol Biol. 2003;63(1):9.1-11. 10.1002/0471142727.mb0901s63 18265332

[15] Nice TJ Strong DW McCune BT. A single-amino-acid change in murine norovirus NS1/2 is sufficient for colonic tropism and persistence. J Virol. 2013;87(1):327-34. 23077309 10.1128/JVI.01864-12 PMC3536416

[16] Zhang J Wang G Ma J. HDAC3 integrates TGF-β and microbial cues to program tuft cell biogenesis and diurnal rhythms in mucosal immune surveillance. Sci Immunol. 2024;9(99):eadk7387. 39331726 10.1126/sciimmunol.adk7387 PMC13337329

[17] Strine MS Alfajaro MM Graziano VR. Tuft-cell-intrinsic and-extrinsic mediators of norovirus tropism regulate viral immunity. Cell Rep. 2022;41(6):111593. 36351394 10.1016/j.celrep.2022.111593 PMC9662704

[18] Kuang Z Wang Y Li Y. The intestinal microbiota programs diurnal rhythms in host metabolism through histone deacetylase 3. Science. 2019;365(6460):1428-34. 31604271 10.1126/science.aaw3134 PMC7158748

[19] Méndez-Ferrer S Lucas D Battista M. Haematopoietic stem cell release is regulated by circadian oscillations. Nature. 2008;452(7186):442-7. 18256599 10.1038/nature06685

[20] Stenzinger M Karpova D Unterrainer C. Hematopoietic-extrinsic cues dictate circadian redistribution of mature and immature hematopoietic cells in blood and spleen. Cells. 2019;8(9):1033. 31491915 10.3390/cells8091033 PMC6769956

[21] Borrmann H McKeating JA Zhuang X. The circadian clock and viral infections. J Biol Rhythms. 2021;36(1):9-22. 33161818 10.1177/0748730420967768 PMC7924106

[22] Nice TJ Baldridge MT McCune BT. Interferon-λ cures persistent murine norovirus infection in the absence of adaptive immunity. Science. 2015;347(6219):269-73. 25431489 10.1126/science.1258100 PMC4398891

[23] Quinnan GV Jr Manischewitz JF. Genetically determined resistance to lethal murine cytomegalovirus infection is mediated by interferon-dependent and-independent restriction of virus replication. J Virol. 1987;61(6):1875-81. 3033318 10.1128/jvi.61.6.1875-1881.1987 PMC254193

[24] Geist LJ Hinde SL. Susceptibility to cytomegalovirus infection may be dependent on the cytokine response to the virus. J Investig Med. 2001;49(5):434-41. 10.2310/6650.2001.33788 11523699

[25] Nadjsombati MS Niepoth N Webeck LM. Genetic mapping reveals Pou2af2/OCA-T1–dependent tuning of tuft cell differentiation and intestinal type 2 immunity. Sci Immunol. 2023;8(83):eade5019. 37172102 10.1126/sciimmunol.ade5019 PMC10308849

